# The Dynamic Change in Plasma Epstein–Barr Virus DNA Load over a Long-Term Follow-Up Period Predicts Prognosis in Nasopharyngeal Carcinoma

**DOI:** 10.3390/v15010066

**Published:** 2022-12-25

**Authors:** Amina Gihbid, Raja Benzeid, Abdellah Faouzi, Imane El Alami, Nezha Tawfiq, Nadia Benchakroun, Karima Bendahhou, Abdellatif Benider, Amal Guensi, Wafa Khaali, Imane Chaoui, Mohammed El Mzibri, Rachida Cadi, Meriem Khyatti

**Affiliations:** 1 Laboratory of Viral Oncology, Institut Pasteur du Maroc, Casablanca 20360, Morocco; 2 Laboratory of Pathophysiology, Molecular Genetics and Biotechnology, Faculty of Sciences Ain Chock, Hassan II University, Casablanca 20100, Morocco; 3 Biology and Medical Research Unit, National Center of Energy, Sciences and Nuclear Techniques, Rabat 10000, Morocco; 4 Laboratory of Medical Virology & BSL-3, Institut Pasteur du Maroc, Casablanca 20360, Morocco; 5 Mohammed VI Center for Cancer Treatment, Ibn Rochd University Hospital, Casablanca 20100, Morocco; 6 Nuclear Medicine Department, Ibn Rochd University Hospital, Hassan II University, Casablanca 10001, Morocco

**Keywords:** circulating EBV DNA load, nasopharyngeal carcinoma, TNM classification, prognostic, biomarker

## Abstract

The current study was designed to investigate the changes in the circulating Epstein–Barr virus DNA load (EBV DNA) at various time points before and after treatment and its clinical significance in nasopharyngeal carcinoma (NPC). A total of 142 patients with NPC were prospectively enrolled in this study. The plasma EBV DNA concentration was measured before and after treatment using qPCR. The prognostic values of the EBV DNA load were analyzed using the Kaplan–Meier and Cox regression tests. Following multivariate analysis, our data showed that high pre-EBV DNA loads were associated with significantly poorer distant metastasis free survival (DMFS) and progression free survival (PFS); detectable end-EBV DNA loads were associated with significantly worse loco-regional recurrence free survival (LRRFS) and PFS, and the detecTable 6 months-post-EBV DNA loads were associated with significantly poorer overall survival (OS), DMFS and PFS (*p* < 0.05). Additionally, combining the pre-EBV DNA load and the stage of the disease, our results showed that patients at stage III-IVA with a low pre-EBV DNA load had similar survival rates as patients at stage II with a low or high pre-EBV DNA load, but had better survival rates than those at stage III-IVA with a high pre-EBV DNA load. Taken together, we showed that the change of the EBV DNA load measured at several time points was more valuable than at any single time point for predicting patients’ survival for NPC. Furthermore, combining the pre-EBV DNA load and the TNM classification could help to formulate an improved prognostic model for this cancer.

## 1. Introduction

Nasopharyngeal carcinoma (NPC) is a squamous cell carcinoma arising from the epithelium of the nasopharynx [1]. Although NPC remains a rare head and neck cancer worldwide, it is particularly endemic in South China, Southeast Asia and North Africa with age-standardized rates ranging from 4 to 25 cases per 100,000 individuals [2,3]. The etiology of NPC is multifactorial, involving host genetic factors, Epstein–Barr virus (EBV) infection and environmental factors [4,5,6].

Currently, radiotherapy (RT) combined with cisplatin-based chemotherapy is the commonly used treatment regimen for patients diagnosed with loco-regionally advanced NPC; RT alone being used to treat limited nasopharyngeal tumors [7]. Generally, clinical observations have revealed that more than 70% of patients present, at diagnosis, a loco-regionally advanced disease with a very poor prognosis, and approximately 20–30% of patients will ultimately develop local or distant recurrence after treatment. NPC prognosis is usually based on tumor and node classification, and is largely affected by the disease stage and treatment approach. This prognosis approach is regularly confronted with the development of a local or distant recurrence [8]. Therefore, the identification of new prognostic factors, as a part of a targeted therapy approach, is of great importance for better management of NPC [9,10].

Accumulating evidence has shown that circulating EBV DNA in NPC patients, derived from nasopharyngeal cancer cells [11], correlates with tumor volume and extension [12,13]. Accordingly, elevated circulating EBV DNA load before NPC onset has been suggested as an interesting biomarker for NPC screening in endemic areas [14]. 

To date, the circulating EBV DNA load before and at the end of treatment has emerged as a promising biomarker for NPC diagnosis and a valuable tool for disease prognosis. Recent studies suggest the EBV DNA load as a potential dynamic biomarker that can change reversibly reflecting NPC tumor progression. Studies from endemic areas for NPC, showed that patients with a detectable EBV DNA load at the end of, and 3-months after, treatment had high rates of loco-regional recurrence and distant metastasis, compared to those with an undetectable EBV DNA load [10,15,16]. However, few studies have evaluated EBV DNA as a dynamic biomarker over a long time period after treatment [17,18]. Thus, the present study was planned to investigate the dynamic change in the plasmatic EBV DNA load before treatment (pre-DNA) and at various time points after treatment (at the end, and 6, 18 and 24 months’ (mo) post-treatment) and its clinical significance in NPC. A risk classification system based on the pre-EBV DNA load and the TNM classification was also evaluated in this study. 

## 2. Materials and Methods

### 2.1. The Study Setting

The study included 142 patients, with histologically confirmed NPC, treated at the Mohammed IV Center for Cancer Treatment of Casablanca between January 2017 and March 2019. All patients were followed until February 2021 or until death. The research protocol was approved by the ethical committee of Ibn Rochd Hospital of Casablanca—Morocco, and written informed consents were obtained from all participants. The characteristics of the NPC patients recruited in this study are described in Table S1.

The pre-treatment evaluation included a complete patient history, physical examination, biological tests, magnetic resonance imaging (MRI) and/or computed tomography (CT) of the nasopharynx and neck, abdominal sonography or CT and/or positron emission tomography (PET)-CT. Detailed epidemiological and clinical characteristics were collected using a direct questionnaire with patients and from the medical records. All patients were staged according to the seventh edition of the American Joint Committee on Cancer (AJCC) staging system based on imaging data. 

All patients were routinely examined at the end of treatment, and then every 3 months during the first year, every 6 months during the second and third year and annually thereafter. Using EDTA tubes, a 10-mL blood sample was collected from each patient before treatment (pre-), at the end of treatment (end-), and at 6, 18 and 24 months after treatment achievement. Due to patient drop-out rates, the blood sampling, which was initially performed for a cohort of 142 NPC patients, was reduced to 75, 51, 26 and 16 patients, at the end of treatment, and at 6, 18 and 24 months after treatment completion, respectively. 

### 2.2. The Quantification of Plasmatic EBV DNA Load

DNA extraction from plasma and circulating EBV DNA quantification were performed as previously described [13]. Briefly, 200 µL of plasma was used to extract DNA using the QIAamp DNA Mini Kit (Qiagen, France). A real-time quantitative polymerase chain reaction (q-PCR) was used to assess circulating EBV DNA following the manufacturer’s recommendations, and results were expressed as International Units /mL (IU/mL). 

### 2.3. The Follow-Up of NPC Patients

All patients were subject to a post-treatment follow-up according to the hospital practice guidelines. Information regarding patient survival was collected through medical records and/or by telephone contact with patients. The overall survival (OS) was defined as the interval from the date of diagnosis to the date of death from any cause or the last follow-up. The loco-regional recurrence-free survival (LRRFS) was defined as the interval from the date of diagnosis to the first evidence of radiological or histological loco-regional recurrence, death from any cause or the last follow-up. The distant metastasis free survival (DMFS) was defined as the interval from the date of diagnosis to the first evidence of radiological distant metastatic lesions, death from any cause or the last follow-up. The progression free survival (PFS) was defined as the time from the diagnosis to progression of the disease, death or the last follow-up (whichever occurred first). 

### 2.4. Statistical Analysis

The statistical analysis was performed with SPSS (version 22.0, Chicago, IL, USA). A receiver operating characteristic (ROC) curve analysis was used to estimate the cut-off values of pre-EBV DNA levels. Life-table estimation was obtained using the Kaplan–Meier method and differences across groups were evaluated with the log-rank test. The Cox proportional hazard model was further used for multivariate analysis, including the following variables: gender, age (≥30 vs. <30 years), T stage (T1–2 vs. T3–4), N stage (N0–1 vs. N2–3), overall disease stage (I-II vs. III-IV) and pre- and post-EBV DNA levels. The results were presented as hazard ratios (HRs) with 95% confidence intervals (CIs). For all tests, the significance level was set at *p* <  0.05.

## 3. Results

### 3.1. The Dynamic Change of Pre- and Post-Treatment Plasma EBV DNA Load

Pre-EBV DNA was detectable in 90.1% of patients (128/142) with a mean viral load of 0.4 UI/mL (range: 0–2.7 × 10^6^ UI/mL). At the end of treatment, EBV DNA was still detected in 16.0% of patients (12/75), with a mean viral load of 142.6 UI/mL (range: 0–3.9 × 10^3^ UI/mL). EBV DNA was detected in 25.5% of patients, 6 months after the end of treatment (13/51) (6-mo- EBV DNA mean: 300.2 UI/mL, range 0–8 × 10^3^ UI/mL); in 15.4% of patients, 18 months after the end of treatment (4/26) (18-mo- EBV DNA mean: 236.9 UI/mL, range: 0–4 × 10^3^ UI/mL); and in only 1 patient, 24 months after treatment achievement (6.25%) (viral load: 312.5 UI/mL) (Table 1). 

Our data further showed that all patients (14/142) with undetectable pre-EBV DNA, maintained undetectable EBV DNA at all post-treatment analyses (end, 6 mo, 18 mo and 24 mo). Furthermore, among the 63/75 (84%) patients with undetectable end-EBV DNA, nine patients had detectable EBV DNA after 6 and/or 18 months of achieving the treatment; the characteristics of these patients are detailed in Table S2 of supplementary data. Of particular interest, the majority of these patients were diagnosed at advanced disease stages: one patient at stage II, two at stage III, three at stage IV-A and one at stage IV-C; and five of them developed disease recurrence during the follow-up. 

### 3.2. The Determination of the Cut-Off Values

The cut-off value of the pre-EBV DNA load, defined using ROC curve analyses, was 4000 UI/mL (Figure S1). This cut-off value was then used to classify NPC patients into low and high pre-EBV DNA groups. After grouping patients based on their pre-EBV DNA levels, 63 patients (44.3%) were in the low pre-EBV DNA load group (<4000 UI/mL) and 79 patients (55.6%) in the high pre-EBV DNA load group (≥4000 UI/mL). Referring to previous studies [10,19], a cut-off value of 0 UI/mL was used for the post-EBV-DNA load to classify patients into undetectable and detectable EBV DNA groups for all post-treatment end points. 

### 3.3. Survival Outcomes (OS and PFS) among Age Subgroups of NPC Patients According to the Plasma Pre and End-EBV DNA Load

Considering that the age incidence curve of NPC in our population shows a bimodal distribution with a significant age peak in the teens and the potential difference in NPC pathogenesis between children and adult patients, we performed a subgroup analysis according to age. As shown in Table 2, poor OS and PFS rates were observed in NPC patients with a high pre-EBV DNA load and a detectable end-EBV DNA load, regardless of the age group of our patients. 

### 3.4. Survival Outcomes According to EBV DNA Load at Various Time Points

**Figure 1:**
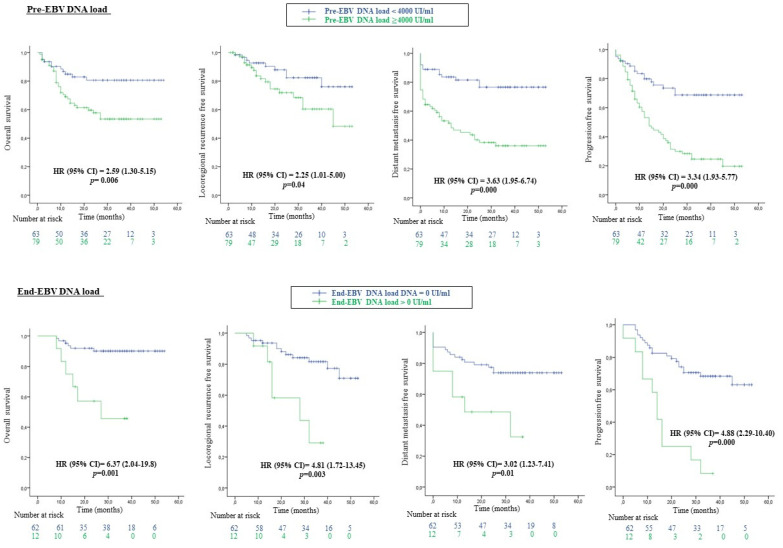
The 4-years’ survival outcomes of patients with NPC according to the EBV DNA load before and at the end of treatment.

**Figure 2:**
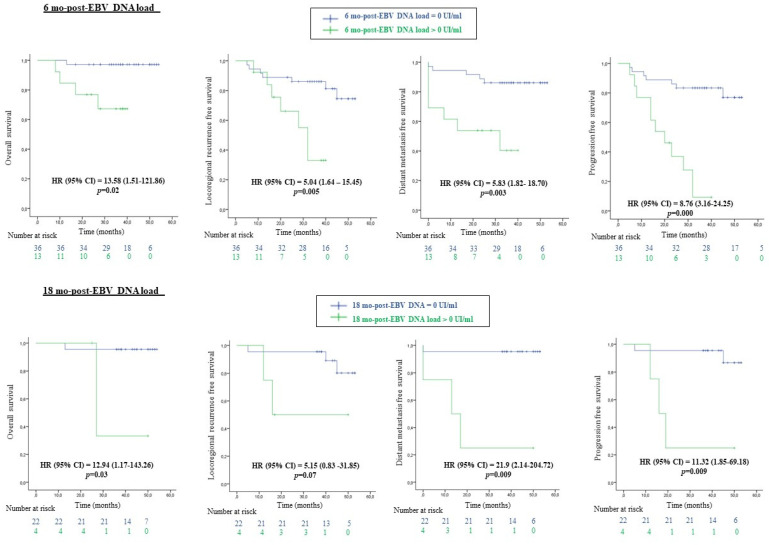
The 4-years’ survival outcomes of patients with NPC according to EBV DNA load 6 and 18 months after treatment.

The association between OS, LRRFS, DMFS and PFS of patients with NPC and EBV DNA load was assessed and reported in Figure 1 and Figure 2. Our results showed that a high pre-EBV DNA load was significantly associated with worse OS (HR CI 95% = 2.59 (1.30–5.15), *p* = 0.006), LRRFS (HR (CI 95%) = 2.25 (1.01–5.00), *p* = 0.04), DMFS (HR (CI 95%) = 3.63 (1.95–6.74), *p* = 0.000) and PFS (HR (CI 95%) = 3.34 (1.93–5.77), *p* = 0.000). Similarly, detectable end-EBV DNA was strongly associated with a high risk of death (HR (95%) = 6.37 [(2.04–19.8), *p* = 0.001], loco-regional recurrence (HR (CI 95%) = 4.81 (1.72–13.45), *p* = 0.003), distant metastasis (HR (CI 95%) = 3.02 (1.23–7.41), *p* = 0.01) and disease progression (HR (CI 95%) = 4.88 (2.29–10.40), *p* = 0.000). 

Patients with an undetecTable 6-mo-EBV DNA load achieved better OS (HR (CI 95%) = 13.58 (1.51–121.86), *p* = 0.02), LRRFS (HR (CI 95%) = 5.04 (1.64–15.45), *p* = 0.005), DMFS (HR (CI 95%) = 5.83 (1.82–18.70), *p* = 0.003) and PFS (HR (CI 95%) = 8.76 (3.16–24.25), *p* = 0.000) compared to patients with a detecTable 6 mo-EBV DNA load. Furthermore, patients with an undetecTable 18 mo-EBV DNA load had significantly higher OS (HR (CI 95%) = 12.94 (1.17–143.26), *p* = 0.03), DMFS (HR (CI 95%) = 21.9 (2.14–204.72), *p* = 0.009) and PFS (HR (CI 95%) = 11.32 (1.85–69.18), *p* = 0.009) rates.

### 3.5. The Subgroup’s Analysis of Dynamic Change in Plasmatic EBV DNA Loads

**Figure 3:**
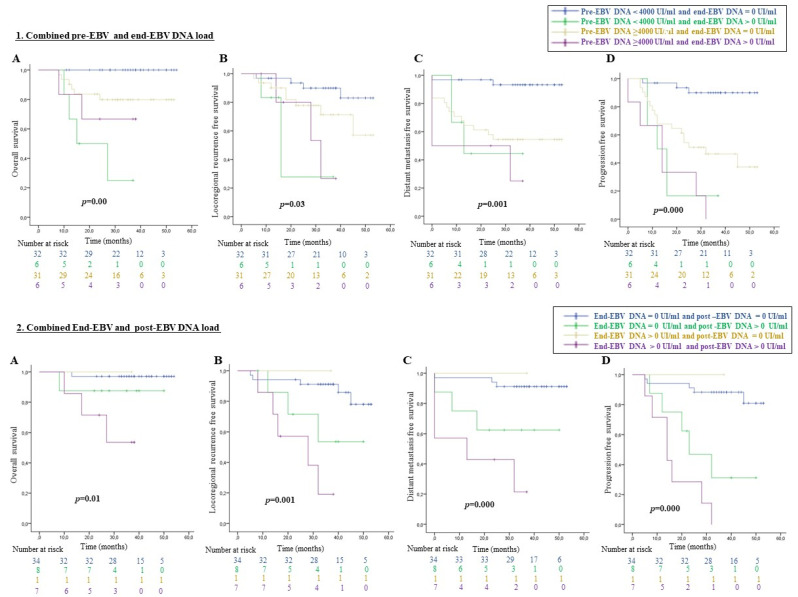
(**A**) Overall survival, (**B**) loco-regional recurrence free survival, (**C**) distant metastasis free survival, (**D**) progression free survival are illustrated for the different subgroups of patients with NPC according to the dynamic change in plasmatic EBV DNA loads.

In the current study, the 75 patients for which EBV DNA tests were performed at both pre- and end-treatment time points, were stratified into four subgroups (Figure 3 (**1**)): subgroup 1 (low pre-EBV DNA and undetectable end-EBV DNA loads, *n* = 32 patients) (42.7%), subgroup 2 (low pre-EBV DNA and detectable end-EBV DNA loads, *n* = 6 patients (8%)), subgroup 3 (high pre-EBV DNA and undetectable end-EBV DNA loads, *n* = 31 patients (41.3%)) and subgroup 4 (high pre-EBV DNA and detectable end-EBV DNA loads, *n* = 6 patients (8%)). Interestingly, subgroup 1 had the highest rates of OS (100%), LRRFS (83.0%), DMFS (93.3%), and PFS (89.9%); followed by subgroup 3, exhibiting OS, LRRFS, DMFS and PFS rates of 71.1%, 71.3%, 54.5% and 37.1%, respectively. Regardless of the pre-EBV DNA load, patients with detectable end-EBV DNA loads (subgroup 2 and 4), presented poorer survival rates, with an OS of 79.0% and 26.7%, SSRLR of 27.8% and 26.7%, SSMD of 44.4% and 25.0% and PFS of 16.7% and 0%, respectively. 

The patients who were subjected to EBV DNA quantification at the end of treatment and at post-treatment times (6 and/or 18 mo), were divided into 4 subgroups: subgroup 1′ (the end-EBV DNA undetectable and post-EBV DNA undetectable, *n* = 34 patients (68%)), subgroup 2′ (the end-EBV DNA undetectable and post-EBV DNA detectable, *n* = 9 patients (18%)), subgroup 3′ (the end-EBV DNA detectable and post-EBV DNA undetectable, *n* = 1 patient (2%)), and subgroup 4′ (the end-EBV DNA detectable and post-EBV DNA detectable, *n* = 6 patients (12%)). The assessment of prognostic values of the end-EBV DNA paired with the post-EBV DNA load (6-mo and 18 mo-EBV DNA load) is depicted in Figure 3 (**2**) and the results showed statistically significant differences for OS, LRRFS, DMFS and PFS rates between the four subgroups (*p* < 0.05). Accordingly, the patients of subgroup 4′ had the worst prognosis with an OS of 53.4%, LRRFS of 19%, DMFS of 21.4% and null PFS rates, followed by those of subgroup 3′ with an OS of 87.5%, LRRFS of 53.6%, DMFS of 62.5 % and PFS of 31.3%. It is also noteworthy that the patients of subgroup 1′ had the best prognosis with the highest OS (97.1%), LRRFS (77.9%), DMFS (91.2 %) and PFS (80.9%) rates. 

### 3.6. The Prognostic Value of the Combined Pre-EBV DNA Load and the TNM Classification

Considering the pre-EBV DNA load as a factor of stratification, patients’ survival analysis (OS and PFS) according to the T, N, M and UICC stages’ categories, clearly showed that the OS and PFS rates were higher in patients with an early tumor (T1) and nodal (N0) and/or overall NPC stages (I–II), regardless of the EBV viral level (Table S3). In patients diagnosed at the advanced tumor (T2–4), nodal (N1–3) and/or overall NPC stages (III–IVA), the OS and PFS rates were significantly higher in those with low pre-EBV DNA loads as compared to those with high pre-EBV DNA loads. 

Table 3 compares the 4-years’ survival rates among subgroups defined by combining the plasma pre-EBV DNA load, TNM classification and UICC stages. Regarding tumor size, our results showed that patients with T2-4, exhibiting low pre-EBV DNA levels had better OS, LRRFS, DMFS and PFS rates compared to those with high pre-EBV DNA loads (82.7% vs. 52.3%, *p* = 0.007; 79.4% vs. 48.4%, *p* = 0.18; 63.5% vs. 35.1%, *p* = 0.00; 68.3% vs. 20.2%, *p* = 0.00, respectively). Similarly, pre-EBV DNA loads can identify high risk subgroups among patients classified as N1–2. In fact, significantly high rates of OS, LRRFS, DMFS and PFS were reported in the N1–2 patients having low pre-EBV DNA loads compared to those with high pre-EBV DNA loads (81.5% vs. 57.1%, *p* = 0.00; 87.7% vs. 43.1%, *p* = 0.02; 68.3% vs. 33.8%, *p* = 0.00 and 73.9% vs. 19.3%, *p* = 0.00, respectively). Patients with N0 had the best survival rates, whereas those with N3 had the worst survival rates, regardless of pre-EBV DNA levels. Notably, our findings highlight that metastatic patients with low pre-EBV DNA loads had significantly better OS, LRRFS and PFS compared to those with high pre-EBV DNA loads (54.5% vs. 15.0%, *p* = 0.00; 100% vs. 66.4%, *p* =0.02; 45.5% vs. 0%, *p* = 0.00, respectively).

Regarding the UICC stages, the patients at stages I–II had the best prognosis, while those at stages IVB had the worst prognosis, regardless of pre-EBV DNA loads. For the patients at stages III–IVA, high pre-EBV DNA loads were found to be associated with poorer OS, LRRFS, DMFS and PFS rates (75.8%, 40.5%, 57.3% and 18.2%, respectively), compared to the patients exhibiting low pre-EBV DNA loads (86.9%, 81.5%, 85.6% and 75.8%, respectively). Interestingly, the patients at stages III–IVA with low pre-EBV DNA load had favorable survival rates and similar prognosis to the patients at stage II (OS = 86.9% vs. 88.9%, LRRFS = 81.5% vs. 81.5%, DMFS = 85.6% vs. 88.9% and PFS = 75.8% vs. 88.9%), respectively. 

### 3.7. The Multivariate Survival Analysis for Survival Endpoints

All factors displaying a significant association in the univariate analysis were further explored in a multivariate analysis, in order to determine if they could be regarded as independent prognostic factors for NPC. Our results indicated that higher pre-EBV DNA loads were independently associated with a poorer DMFS and PFS (*p* = 0.01 and 0.02, respectively). Moreover, the end-EBV DNA load was an independent prognostic factor for the LRRFS and PFS (*p* < 0.05); and the 6mo-post-EBV DNA load was an independent prognostic factor for the OS, DMFS and PFS (*p* = 0.02, 0.04 and 0.004, respectively) (Table 4). The pre- and end-EBV DNA load combination was found to predict LRRFS, DMFS and PFS (*p* = 0.006, 0.03 and 0.001, respectively), while the end- and post- EBV DNA load combination was found to predict PFS (*p* = 0.005).

## 4. Discussion

During the last decade, increasing interest has been directed toward pre- and end-EBV DNA loads as promising biomarkers for early detection, diagnosis and prognosis of NPC [20,21]. In this study, we showed that the plasmatic pre-EBV DNA was detected in 90.1% of NPC patients with a mean viral load of 0.4 UI/mL, which is consistent with the widely reported prevalence rates ranging from 53% to 96% [22]. Furthermore, and as expected, the frequency of circulating EBV DNA positivity and levels of viral load decreased considerably at the end of treatment to 16.0% and to a mean viral load of 142.6 UI/mL, respectively. However, although the clinical value of EBV DNA load testing before and after treatment has been well documented and discussed, most follow-up studies have focused on only a few time points after treatment completion (at the end of treatment and 3 months after treatment). According to the available data, EBV DNA clearance from the plasma of NPC patients occurs rapidly after treatment, generally after the two first weeks of RT, and any increase after the end of treatment is typically a sign of disease recurrence [10,16,23,24,25]. The EBV DNA can recur 3 months after the end of treatment, highlighting the need for long-term monitoring of the EBV DNA load dynamic in NPC patients [15]. In this context, our results showed that 25.5% (mean = 300.2 UI/mL), 15.4% (mean = 236.9 UI/mL) and 6.25% (mean = 312.5 UI/mL) of patients had detectable EBV DNA in their plasma at 6, 18 and 24 months’ post-treatment, respectively. These results agree with previous evidence suggesting that EBV DNA in the plasma of NPC patients is derived from apoptotic and necrotic tumor cells and may reflect the tumor’s growth and decline [11]. 

Considering that for NPC, the OS, LRRFS, DMFS and PFS are the commonly used endpoints for patient follow-up and treatment efficiency evaluation, the association between the EBV DNA load and these four survival parameters was investigated. Our results indicated that the NPC patients with high pre-EBV DNA loads and detectable end-EBV DNA loads have poor survival rates, regardless of the age group. Our findings further revealed that a higher pre-EBV DNA load (≥ 4000 UI/mL) was independently associated with poorer DMFS and PFS (*p* < 0.05); and a detectable end-EBV DNA load was an independent prognostic factor for worse LRRFS and PFS (*p* < 0.05) in NPC. Furthermore, by using the multivariate prognostic model, our data showed that the pre- and end-EBV DNA load combination predicts, independently, the LRRFS, DMFS and PFS (*p* < 0.05). These results concur with data from several Chinese studies, supporting the pre-and end-EBV DNA load as a good prognostic biomarker in NPC [26,27]. During follow-up, we revealed that patients with detecTable 6-mo and 18-mo-EBV DNA loads had a higher risk of mortality, loco-regional recurrence, distant metastasis and disease progression (more than 12, 5, 5 and 8 fold, respectively), than those with undetecTable 6-mo and 18-mo levels of EBV DNA. Additionally, end- and post- EBV DNA load combination predict, independently, the PFS (*p* = 0.005) in the multivariate analysis. These patients’ poor survival outcomes may be linked to the persistence of residual tumor cells that can escape early endoscopic and radiological post-treatment evaluations, as well as chemo-radiotherapy sensitivity that varies among individuals even at the same stage of the disease. In this context, Twu et al. have suggested the benefit of providing additional adjuvant chemotherapy for patients with persistently detectable post-EBV DNA loads after RT, plus induction/concurrent chemotherapy, which would significantly reduce distant failures and improve the overall survival of these patients [28].

Currently, and according to most studies, the patients with detectable end-EBV DNA loads are at an extremely high risk of loco-regional and distant recurrences, whereas the patients with undetectable EBV DNA after treatment have a good prognosis. In our study, the end-EBV DNA load was undetectable, but reemerged 6 and/or 18 mo later in the plasma of nine patients, of whom five subsequently developed loco-regional and/or distant failure. This reappearance of plasmatic EBV DNA after its clearance, which may precede clinical signs of the disease recurrence, could be an accurate early predictor of disease recurrence [15]. We therefore believe that post- treatment EBV DNA quantification is of significant relevance in post-treatment evaluation and can improve the follow-up evaluation of patients, adding value to routine endoscopy and radiology examination. Meanwhile, a specific algorithm for introducing post-EBV DNA quantification in NPC management should be proposed. Moreover, patients with a 6-mo and/or 18-mo positive post-EBV DNA load and a negative post-treatment endoscopy or normal CT/MRI should be monitored more closely, preferably with biopsy endoscopy and PET/CT; especially in the presence of local inflammation and the fibrotic side effects resulting from RT. 

Concurring with previous studies [29,30], we observed that the patients with undetectable pre-EBV DNA loads maintained an undetectable end-, 6-mo and 18-mo-EBV DNA load. A possible explanation for this might be that this group of patients may have non-EBV origin tumors. There are, however, other possible explanations. In fact, the absence of, or a low, detectable plasma EBV DNA may also be due to tumor heterogeneity in the EBV copy number that ranged from 2 to 137 copies EBV/cell [31]. Additionally, other studies have reported variability in copies of the promoter Wp within each genome in NPC. In this field, Nicolls et al. found either a low or the absence of the expression of EBER in tumors of patients with undetectable plasma EBV DNA, as compared to those with a detectable EBV DNA load; suggesting that the EBV genome may be poorly incorporated in the tumor cells in this subgroup of patients leading to impaired expression of the EBER and plasma EBV DNA production [30]. 

The results of the present study, as well as those of subsequent studies, confirm the great interest of the EBV DNA load in the management of NPC [13,32]. However, the most important challenges remain as the use of the EBV DNA load in patients’ staging and the risk stratification before, during and after chemo-radiotherapy. Accordingly, and rather than replacing TNM staging, few studies suggest that pre-EBV DNA loads may be used to complement and refine this conventional approach to better assess patient prognosis [35,36]. We previously reported a significant association between pre-EBV DNA load, TNM classification, overall stage and OS [13]. In the present study, we showed that the pre-EBV DNA load is a promising biomarker, able to identify among patients at the same or different disease stages, a subgroup with a high risk of recurrence or treatment failure. Indeed, the data of the present study revealed that patients at stage III-IVA with a low pre-EBV DNA load had comparable survival rates to patients at stage II with low or high pre-EBV DNA loads, but had better survival rates than those at stage III-IVA with a high pre- EBV DNA load. These results are globally aligned with those reported by Zhang et al. that have suggested a segregation of disease stages into four different risk groups with regards to the pre-EBV DNA load [33]: (1) very low risk, stage I regardless of the pre-EBV DNA load; (2) low risk, stages II, III, IVa–b with low EBV DNA loads; (3) intermediate risk, stages II–III with high EBV DNA loads; (4) high risk, stages IVa–b with high EBV DNA loads. More recently, two studies proposed refining the eighth edition of the AJCC/UICC TNM staging system by incorporating the pre-EBV DNA load. This refinement should help to improve the prognostic performance of the AJCC/UICC TNM staging system and contribute to the development of individualized treatment strategies for NPC, for better management of this disease [34,35]. In this field, a recent study proposed a validated recursive-partitioning analysis (RPA) model to classify NPC patients based on their end-EBV DNA levels and the TNM stage, and showed that the combination of the circulating end-EBV DNA and the TNM stage improved the risk stratification of NPC. Interestingly, the RPA model (c-index 0.712) showed better risk discrimination compared to the TNM stage (0.604) or the end-EBV DNA alone (0.675) [3]. 

The present study is clearly informative and highlights the interest of integrating EBV DNA loading in the management of NPC prognosis and disease risk stratification. This should, however, be interpreted in the context of its limitations: (1) the prospective nature of the study involved a small number of patients; (2) the low number of patients with a post-EBV DNA load, owing to different constraints including, patients who were lost to follow up; (3) the middle follow-up time (4 years). Further research including a larger number of patients and a longer follow-up period will undoubtedly refine the results of this study.

## 5. Conclusions

The results showed strong evidence that EBV DNA load testing is a promising dynamic and a minimally invasive biomarker for prognosis and follow-up of the juvenile and adult form of NPC. Regardless of the time point, patients with a high pre-EBV DNA load had poorer survival rates, and persistently high or detectable post-EBV DNA levels might be a good indicator of local, regional or distant tumor recurrence. The EBV DNA load is, therefore, a good biomarker to complete conventional approaches for better management of NPC. 

## Figures and Tables

**Table 1:** The prevalence of EBV DNA and EBV DNA levels in the plasma of patients with NPC at various time points.

	Number of Patients	Prevalence of EBV DNA	Mean EBV DNA Levels (UI/mL)
**Pre-EBV DNA load**	142	90.1% (128/142)	0.4 [0–2.7 × 10^6^]
**End-EBV DNA load**	75	16.0% (12/75)	142.6 [0–3.9 × 10^3^]
**6-mo-EBV DNA load**	51	25.5% (13/51)	300.2 [0–8 × 10^3^]
**18-mo-EBV DNA load**	26	15.4% (4/26)	236.9 [0–4 × 10^3^]
**24-mo-EBV DNA load**	16	6.25% (1/16)	312.5 [0–5 × 10^3^]

**Table 2:** The 4-years’ survival rates (OS and PFS) among age subgroups of NPC patients according to plasma EBV DNA load before and at the end of treatment.

		OS Rates (%)		PFS Rates (%)		OS Rates (%)		PFS Rates (%)	
		Pre-DNA EBV Load (UI/mL)				End-DNA EBV Load (UI/mL)			
**Group of Age**	**Number and Percentage of Patients**	<4000	≥4000	<4000	≥4000	<0	≥0	<0	≥0
[12–21]	22/142 (15.5%)	81.8	69.3	81.8	60.6	90.0	50.0	81.8	50.0
[22–32]	13/142 (9.2%)	100	50	77.8	0.00	81.8	-	62.3	0.00
[33–42]	21/142 (14.8%)	85.7	66.7	52.5	32.4	100	66.7	65.7	0.00
[43–52]	36/142 (25.4%)	87.5	56.6	76.6	10.7	100	50.0	61.9	0.00
[53–62]	35/142 (24.6%)	58.4	58.6	49.6	24.2	80.4	0.00	47.8	0.00
[63–72]	14/142 (9.9%)	75.0	0.00	75.0	0.00	0.00	-	0.00	-
>72	1/142 (0.7%)	-	0.00	-	0.00	-	-	-	-

**Table 3:** Comparisons of the 4-years’ survival rates among subgroups of NPC patients defined by the combination of plasma pre-EBV DNA, TNM classification and UICC Stages.

	Number of Patients	4-Years OS	*p* Value	4-Years LRFS	*p* Value	4-Years DMFS	*p* Value	4-Years PFS	*p* Value
**Tumor Classification**									
T1-low * or high ** EBV	11/142	72.7%	0.007	70.0%	0.18	63.6%	0.00	54.5%	0.000
T 2,3,4-low EBV	54/142	82.7%		79.4%		63.5%		68.3%	
T 2,3,4- high EBV	77/142	52.3%		48.4%		35.1%		20.2%	
**Lymph Node Status**									
N0-low EBV or high	11/142	100%	0.00	75.0%	0.02	100%	0.00	90%	0.00
N1,2-low EBV	46/142	81.5%		87.7%		68.3%		73.9%	
N1,2-High EBV	58/142	57.1%		43.1%		33.8%		19.3%	
N3- low or high EBV	27/142	42.8%		53.7%		24.3%		16.7%	
**Metastasis Status**									
M0-low or high EBV	96/142	82.0%	0.00	64.0%	0.02	73.4%	0.00	55.9%	0.00
M1-low EBV	11/142	54.5%		100%		0.00%		45.5%	
M1-EBV high	35/142	15.0%		66.4%		0.00%		0.00%	
**UICC Stages**									
I- low or high EBV	4/142	100%	0.40	75.0%	0.01	100%	0.05	100%	0.001
II-low or high EBV	14/142	88.9%		100%		88.9%		88.9%	
III, IVA- low EBV	35/142	86.9%		81.5%		85.6%		75.8%	
III, IVA-high EBV	32/142	75.8%		40.5%		57.3%		29.8%	
IVB- low or high EBV	11/142	70.1%		26.5%		58.9%		18.2%	

Low * EBV: pre-EBV DNA load < 4000 UI/mL; high ** EBV: pre-EBV DNA load ≥ 4000 UI/mL.

**Table 4:** Multivariate Cox regression analyses for the studied survival endpoints according to the EBV DNA load.

	OS		LRRFS		DMFS		PFS	
	Multivariate Analyses (HR (95% CI))	*p* Value	Multivariate Analyses (HR (95% CI))	*p* Value	Multivariate Analyses (HR (95% CI))	*p* Value	Multivariate Analyses (HR (95% CI))	*p* Value
**Pre-EBV DNA load**	0.87 (0.24–3.13)	0.83	0.99 (0.33–2.97)	0.99	4.45 (1.40–14.14)	0.01	2.93 (1.15–7.44)	0.02
**Post-EBV DNA load**	4.45 (0.89–22.15)	0.06	4.18 (1.28–13.61)	0.01	2.58 (0.95–6.95)	0.05	3.96 (1.75–8.92)	0.001
**6 mo-post-EBV DNA load**	29.10 (1.47–575.00)	0.02	2.29 (0.67–7.81)	0.18	4.12 (1.05–16.12)	0.04	5.60 (1.75–17.87)	0.004
**18 mo-post-EBV DNA load**	4.79 (0.40–57.28)	0.21	2.73 (0.33–22.36)	0.34	8.35 (0.83–83.43)	0.07	8.04 (0.80–80.74)	0.07
**Pre and end- EBV DNA load**	-	0.10	-	0.006	-	0.03	-	0.001
**End and post- EBV DNA load**	-	0.52	-	0.18	-	0.07	-	0.005

## Data Availability

Not applicable.

## Supplementary Materials

The following supporting information can be downloaded at: https://www.mdpi.com/article/10.3390/v15010066/s1, Figure S1: The time-dependent receiver operating characteristic curve analysis of OS prediction of NPC patients based on the pre-EBV DNA load.; Table S1: The socio-economic and clinical features of NPC patients recruited in the study (*n* = 142); Table S2: Characteristics of patients with an undetectable end-EBV DNA load and a detecTable 6 (and/or)18-months post-EBV DNA load; Table S3: The 4-years’ survival rates’ (OS and PFS) comparisons between subgroups of patients according to the pre-EBV DNA load and TNM classification.
