# Supplementary material for: The Dynamic Change in Plasma Epstein–Barr Virus DNA Load over a Long-Term Follow-Up Period Predicts Prognosis in Nasopharyngeal Carcinoma
Source: Viruses. 2022 Dec 25;15(1):66. doi: 10.3390/v15010066 (PMC9865665; doi:10.3390/v15010066)
Supplement: Supplementary file 1 [file viruses-15-00066-s001.zip › viruses-2046698-supplementary.pdf]

Dynamic Change in Plasma Epstein–Barr virus DNA Load Over a Long Term Follow-up Period Predicts Prognosis in Nasopharyngeal Carcinoma

Supplementary Data

Amina Gihbid[1,2], Raja Benzeid[3], Abdellah Faouzi[4], Imane El Alami[1], Nezha Tawfiq[5], Nadia Benchakroun[5], Karima Bendahhou[5], Wafaa Khaali[1], Abdellatif Benider[5], Amal Guensi[6], Imane Chaoui[3], Mohammed El Mzibri[3], Rachida Cadi[2] & Meriem Khyatti[1]

1 Laboratory of Viral Oncology, Institut Pasteur du Maroc, Casablanca, Morocco
2 Laboratory of Pathophysiology, Molecular Genetics and Biotechnology, Faculty of Sciences Ain Chock, Hassan II University, Casablanca, Morocco
3 Biology and Medical Research Unit, National Center of Energy, Sciences and Nuclear Techniques, Rabat, Morocco
4 Laboratory of Medical Virology & BSL-3, Institut Pasteur du Maroc, Casablanca, Morocco
5 Mohammed VI Center for Cancer Treatment, Ibn Rochd University Hospital, Casablanca, Morocco
6 Nuclear Medicine Department, Ibn Rochd University Hospital, Hassan II University, Casablanca, Morocco

***Corresponding authors:**

Pasteur Institute of Morocco, 1 Place Louis Pasteur, 20360 Casablanca, Morocco

Phone: +212 5 22 43 44 57 / **Fax:** +212 5 22 26 09 57

Email: meriem.khyatti@pasteur.ma

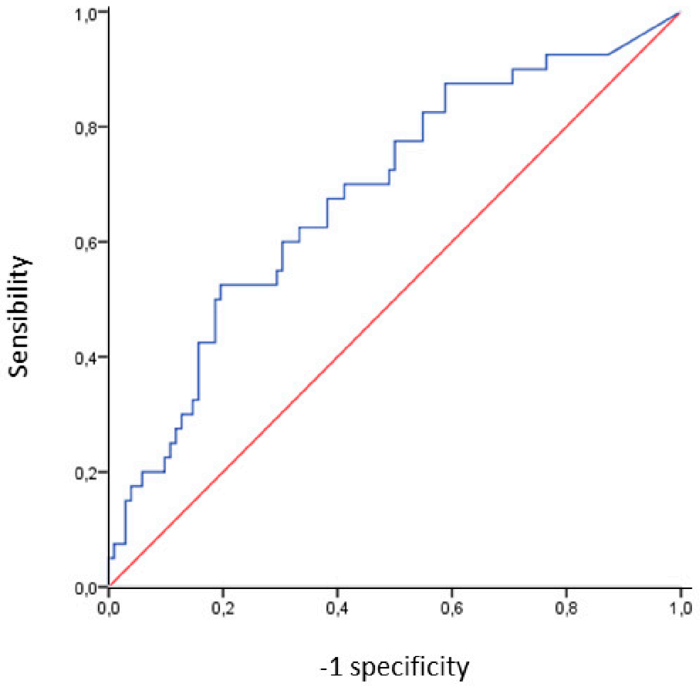

Figure S1: Time-dependent receiver operating characteristic curve analysis of OS prediction of NPC patients based on the pre-EBV DNA load. The area under the curve was 0.67 (p=0.00, 95% CI 0.59–0.79), and 4000 UI/ml was determined as the pre-EBV DNA load best cutoff value for survival prediction.

Table S1: Socio-economic and clinical features of NPC patients recruited in the study (n=142)

Characteristics	Number of cases	%
**Age**		
[12-21]	22/142	15.5
[22-32]	13/142	9.2
[33-42]	21/142	14.8
[43-52]	36/142	25.4
[53-62]	35/142	24.6
[63-72]	14/142	9.9
>72	1/142	0.7
**Gender**		
Female	53/142	37.3
Male	89/142	62.7
**TABAC**		
No	115/142	81.0
Yes	27/142	19.0
**ALCOOL**		
No	123/142	86.6
Yes	19/142	13.4
**Histological type**		
Keratinizing squamous cell carcinoma	1/142	0.7
Non-keratinizing differentiated carcinoma	7/142	4.9
Non-keratinizing undifferentiated carcinoma	131/142	92.3
Others	3/142	2.1
**TNM classification**		
T1-T2	34/142	23.9
T3-T4	108/142	76.1
N0-N1	84/142	33.8
N2-N3	94/142	66.2
M0	96/142	67.6
M1	46/142	32.4
**Stage of the disease**		
I	3/142	2.1
II	14/142	9.9
III	33/142	23.2
IV	92/142	64.8

Table S2: Characteristic of patients with undetectable end-EBV DNA load and detectable 6 (and/or)18-months post-EBV DNA load

**Code of patient**	**22**	**27**	**42**	**46**	**67**	**87**	**90**	**129**
**Age**	51	12	53	44	45	58	34	39
**Gender**	Male	Male	Male	Female	Male	Female	Female	Male
**TNM classification**	T4N2M0	T4N1M0	T3N1M1	T2N1M0	T1N2M0	T4N1M0	T3N2M0	T2N3M0
**Stage of the disease**	IVA	IVA	IVC	II	III	IVA	III	IVB
**Therapy**	NCT*+ RT**	NTC+RCC***	NTC+RCC	RCC	NTC+RCC	NTC+RCC	NCT+RT	NCT+RT
**Pre-EBV DNA load (UI/ml)**	60126	6387	18741	5361	624	12774	72213	669
**End-EBV DNA load (UI/ml)**	0	0	0	0	0	0	0	0
**6- months post-EBV DNA load (UI/ml)**	0	73.2	0	0	877	537	700	169.6
**18- months post-EBV DNA load (UI/ml)**	4000	-	500	9.8	-	-	-	-
**Disease failure**	Local failure and distant metastasis	Distant metastasis	Distant metastasis	Absent	Absent	Locoregional failure	Absent	Locoregional failure
**Time to declaration (month)**	17	7	23	-	-	32	-	20
**Outcome at last time of follow-up**	Alive	Dead	Alive	Alive	Alive	Alive	Alive	Alive
**Follow-up time (month)**	25	8	39	50	40	35	22	28

*NCT: neoadjuvant chemotherapy; ** RT: radiotherapy; *** RCC: concomitant chemoradiotherapy

Table S3: 4-years survival rates (OS and PFS) comparisons between among subgroups of patients according to pre-EBV DNA load and TNM classification

	**OS rates***		**PFS rates***	
	**LOW pre-EBV DNA load (< 4000 UI/ml)**	**High pre-EBV DNA load (≥ 4000 UI/ml)**	**LOW pre-EBV DNA load (< 4000 UI/ml)**	**High pre-EBV DNA load (≥ 4000 UI/ml)**
**Tumor classification**				
**T1 (13)**	88.9%	100%	66.7%	50.0%
T2 (21)	77.8%	43.8%	56.8%	29.2%
T3 (46)	82.6%	48.9%	69.8%	12.3%
T4 (62)	86.3%	54.0%	75.1%	20.7%
**Lymph node status**				
N0 (11)	100%	100%	85.7%	100%
N1 (37)	88.8%	76.2%	77.9%	34.2%
N2 (63)	75.9%	54.2%	70.6	13.1%
N3 (31)	55.6%	30.5%	33.3%	0.00%
**Metastasis status**				
M0	86.0%	77.3%	73.7%	36.7%
M1	54.5%	15.0%	45.5%	0.00%
**Stage of the disease**				
I (3)	100%	100%	100%	-
II (14)	83.3%	100%	83.3%	100%
III (33)	81.9%	76.9%	75.5%	24.3%
IVA (36)	93.8%	76.5%	79.1%	37.6%
IVB (11)	75.0%	64.3%	25.0%	0.00%
IVC (46)	54.5%	14.8%	45.5%	0.00%

Abbreviations: OS: overall survival; PFS: progression-free survival.

*Survival rates were calculated using the Kaplan-Meier method.

Low risk group Middle risk group High risk group
